# Exosomes derived from mouse vibrissa dermal papilla cells promote hair follicle regeneration during wound healing by activating Wnt/β-catenin signaling pathway

**DOI:** 10.1186/s12951-024-02689-w

**Published:** 2024-07-19

**Authors:** Yage Shang, Mengyang Li, Lixia Zhang, Chao Han, Kuo Shen, Kejia Wang, Yan Li, Yue Zhang, Liang Luo, Yanhui Jia, Kai Guo, Weixia Cai, Jian Zhang, Xujie Wang, Hongtao Wang, Dahai Hu

**Affiliations:** Department of Burns and Cutaneous Surgery, Xijing Hospital, Air Force Medical University, Xi’an, China

**Keywords:** Wound healing, Hair follicle regeneration, DPC-Exos, Fibroblasts, Wnt/β-catenin signaling pathway

## Abstract

Hair follicle (HF) regeneration during wound healing continues to present a significant clinical challenge. Dermal papilla cell-derived exosomes (DPC-Exos) hold immense potential for inducing HF neogenesis. However, the accurate role and underlying mechanisms of DPC-Exos in HF regeneration in wound healing remain to be fully explained. This study, represents the first analysis into the effects of DPC-Exos on fibroblasts during wound healing. Our findings demonstrated that DPC-Exos could stimulate the proliferation and migration of fibroblasts, more importantly, enhance the hair-inducing capacity of fibroblasts. Fibroblasts treated with DPC-Exos were capable of inducing HF neogenesis in nude mice when combined with neonatal mice epidermal cells. In addition, DPC-Exos accelerated wound re-epithelialization and promoted HF regeneration during the healing process. Treatment with DPC-Exos led to increased expression levels of the Wnt pathway transcription factors β-catenin and Lef1 in both fibroblasts and the dermis of skin wounds. Specifically, the application of a Wnt pathway inhibitor reduced the effects of DPC-Exos on fibroblasts and wound healing. Accordingly, these results offer evidence that DPC-Exos promote HF regeneration during wound healing by enhancing the hair-inducing capacity of fibroblasts and activating the Wnt/β-catenin signaling pathway. This suggests that DPC-Exos may represent a promising therapeutic strategy for achieving regenerative wound healing.

## Graphical Abstract

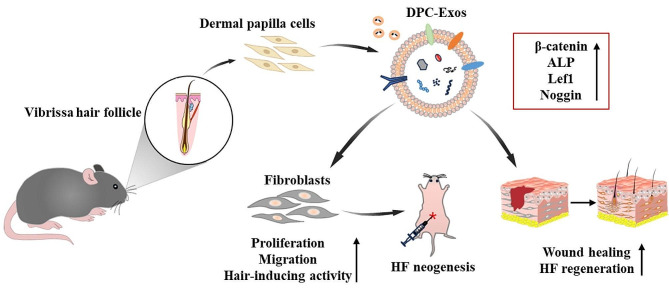

## Introduction

Wound healing is a crucial physiological process essential for restoring skin integrity following injury. Hair follicles (HFs), miniature organs situated in the dermal layer of the skin, play a critical role in human health, contributing to skin metabolism, thermoregulation, sensory perception, and social interactions [1, 2]. In postpartum humans, hair loss due to deep skin injuries often does not regenerate, which not only affects skin function and aesthetics but also causes pain and distress for patients [3, 4]. HF regeneration is paramount in reestablishing normal skin function and minimizing scar formation during the wound healing [5, 6]. While numerous methods have been devised to enhance wound healing outcomes, promoting HF regeneration during this process remains a significant challenge.

Fibrotic scarring and regenerative healing are often considered opposing trajectories in wound healing, with fibroblasts playing a crucial role in determining in determining wound healing outcomes [7–9]. While fetal and neonatal mouse dermal fibroblasts retain the capacity to induce HF neogenesis, this hair-inducing ability is lost in adult fibroblasts [10, 11], contributing to the prevalence of scarring over regenerative healing in adult wounds. Extensive research has been conducted to enhance the hair-inducing capacity of adult dermal fibroblasts, aiming to promote HF regeneration. Ma, Y et al. [12] demonstrated that specific small molecules could induce both the L929 fibroblast cell line and mouse dermal fibroblasts to adopt dermal-papilla-cell-like characteristics, conferring hair-forming ability upon adult mouse fibroblasts. Similarly, Xie et al. [13]. reported that a 3D cultivation system employing hydrogel microcapsules could induce fibroblasts to differentiate into dermal papilla cell-like cells. Despite restoring the fibroblasts trichogenecity and inducing HF neogenesis, these methods may not be readily translatable to wound treatment due to concerns regarding safety and feasibility. With the burgeoning field of regenerative medicine, mesenchymal stem cell-derived exosomes have been considered a promising therapeutic modality for tissue regeneration [14–16].

HF consists of epithelial and mesenchymal components and undergoes cyclical process of active growth (anagen), degeneration (catagen), and quiescence (telogen) [17]. As the smallest organ capable of complete regeneration in the body, HF is extensively utilized in regenerative medicine research. HF morphogenesis relies on the epithelial–mesenchymal interaction (EMI), with dermal papilla cells (DPCs) as a inducible mesenchymal component to drive HF formation [18, 19]. As the signaling hub of hair follicles, DPCs activate the proliferation and differentiation of hair follicle epithelial cells through paracrine mechanisms, with exosomes acting as crucial mediators for this function [20, 21]. Exosomes are extracellular vesicles, ranging from 30 to 150 nanometers in diameter, which comprise proteins, lipids, and nucleic acids, playing a critical role in intercellular communication [22]. Studies have demonstrated that exosomes derived from DPCs (DPC-Exos) can stimulate the proliferation and differentiation of hair follicle stem cells [23], hair matrix cells [24], and outer root sheath cells [25], thereby promoting HF growth, regeneration, and hair cycles.

Considering the role of DPC-Exos in promoting HF regeneration, we evaluated their potential to enhance HF regeneration during wound healing. While the effects of DPC-Exos have been extensively studied, their effect on fibroblasts in wound healing remains unexplored. To address this, we conducted a series of in vitro and in vivo experiments to appraise and verify the effect of DPC-Exos on fibroblasts during wound healing. Our findings indicate that DPC-Exos can stimulate fibroblasts proliferation and migration of fibroblasts, as well as enhance their hair-inducing capabilities. Hair reconstitution assay demonstrated that fibroblasts treated with DPC-Exos could induce HF neogenesis in nude mice when combined with neonatal mice epidermal cells. Moreover, DPC-Exos accelerated wound re-epithelialization and facilitated HF regeneration during wound healing. The involvement of the Wnt/β-catenin signaling pathway in both fibroblasts and wound healing was confirmed through the use of a Wnt pathway inhibitor. Collectively, these results demonstrate that DPC-Exos can promote HF regeneration during wound healing by activating fibroblasts and the Wnt/β-catenin signaling pathway.

## Materials and methods

### DPCs isolation

Dermal papilla(DP) tissues were obtained from the vibrissa follicles of 6-week-old male C57BL/6J mice utilizing microdissection combined with collagenase digestion as previously described [26–28]. In brief, after euthanizing mice, the vibrissa pads ware excised. With the dermal side positioned under a microscope to fully expose the hair follicles, the hair bulb was transected beneath the lower portion of the HF. All hair bulbs were then collected and subjected to digestion in 0.2% type I collagenase (Gibco, Grand Island, USA) at 37 °C for 30 min. The resulting DP spheroids were resuspended in high-glucose DMEM (Gibco, USA) supplemented with 20% FBS (Corning, USA) and 1% penicillin/streptomycin, and then cultured in an incubator maintained at 37 ℃ with 5% CO_2_. By day 7, DPCs exhibited aggregative growth behavior around the DP spheroids. Subcultured DPCs were then cultured in DMEM/F12 (Gibco) supplemented with 10% FBS and 1% penicillin/streptomycin, with passages conducted every 3–4 days. DPCs from passages 3 to 6 were utilized for following assay.

### Flow cytometry and ALP staining of DPCs

The expression of specific markers in DPCs was evaluated utilizing flow cytometry. Passage 4 DPCs were incubated with fluorescence-conjugated antibodies targeting Versican (ab311818, Abcam, 1:200), Sox2 (ab93689, Abcam, 1:200), β-catenin (8480T, CST, 1:200), and ALP (4060T, CST, 1:100). Then, the cells were analyzed by a flow cytometer (BD FACSAria™ III system; USA). The alkaline phosphatase (ALP) activity of DPC was detected by an alkaline phosphatase assay kit (P0321S, Beyotime, China). DPCs were incubated with NBT/BCIP solution, and dark grey staining indicated positive ALP activity. Images were acquired utilizing an Olympus FSX100 microscope.

### Osteoblastic and adipogenic differentiation of DPCs

Passage 4 DPCs were seeded in six-well plates until they reached approximately 80–90% confluency. The culture medium was then removed and replaced with either osteogenic differentiation medium for 3 weeks or adipogenic differentiation medium for 2 weeks. Following the induction of differentiation, DPCs were fixed with 4% paraformaldehyde and stained with Alizarin Red S or Oil Red O to detect the results of osteoblastic and adipogenic differentiation, respectively. Images were observed utilizing an Olympus FSX100 microscope.

### Isolation and identification of DPC-Exos

P3 to P6 DPCs were cultured in 100-mm dishes until they reached 80% confluence. At this point, the culture medium was replaced with DMEM/F12 containing exosome-free FBS. After 48 h cultured at 37 ℃ with 5% CO_2_, the DPC culture supernatants were collected for exosome isolation utilizing differential centrifugation and ultracentrifugation methods [29]. The supernatants were subjected to centrifugation at 300×*g* for 10 min, 2,000×*g* for 10 min, and 10,000×*g* for 30 min to remove cell debris and middle-large extracellular vesicles. Then, the supernatants were ultracentrifuged at 100,000×*g* for 70 min. The resulting pellets were thoroughly washed with cold PBS and ultracentrifuged again for purification and enrichment. Finally, the exosome pellets were resuspended in PBS (200 µL PBS for 200 mL supernatant), and stored at -80 ℃.

The morphology and size distribution of DPC-Exos were characterized utilizing transmission electron microscopy (TEM) and nanoparticle tracking analysis (NTA), respectively. Western blot was employed to detect exosomal protein markers. Prior to use, DPC-Exos were sterilized by filtration through a 0.22-µm filter to remove bacteria. Following a 24-h incubation with the PKH26-labeled DPC-Exos, fibroblasts were fixed with 4% paraformaldehyde, and nuclei were counterstained with DAPI. Imaging was performed utilizing an Olympus FSX100 microscope.

### Fibroblast isolation and culture

Primary fibroblasts were isolated from the dorsal skin of 6-8-week-old male C57BL/6J mice as previously described [12, 30]. Briefly, following hair removal, mice were sterilized with 75% alcohol and rinsed three 3 times with PBS. Dosal skin was harvested and subjected to overnight incubation in a 0.25% dispase-trypsin solution at 4 °C. The epidermal layer was carefully removed, leaving only the dermis. The dermis was cut into fragments and digested with 0.2% type I collagenase at 37 °C for 1 h, followed by further digestion with 0.25% trypsin-EDTA at 37 °C for 10 min. The digestion process was terminated by the addition of serum-containing medium. The resulting cell suspension was filtered through a 100-µm mesh to remove debris. The filtrate was then centrifuged at 300×*g* for 5 min, and the resulting cell pellet was resuspended and plated in culture flasks. Cells were cultured in low-glucose DMEM supplemented with 10% FBS and 1% penicillin/streptomycin in an incubator with 5% CO_2_ at 37 ℃. Fibroblasts at passage 4 were utilized for the following experiments.

### Cell proliferation assay

The proliferation of fibroblasts was measured utilizing the CCK-8 cell proliferation assay and the EdU staining assay. For the CCK-8 assay, fibroblasts were seeded into 96-well plates at a density of 5 × 10^3^ cells/well. Varying concentrations of DPC-Exos (0, 10, 20, and 40 µg/mL) were introduced to the wells at 0, 24, and 36 h following cell adherence. Each group consisted of six replicate wells. After 48 h of incubation, 10 µl of CCK-8 reagent (AR1160-500, Boster, China) was added to each well, followed by a 2 h incubation at 37 °C. Absorbance readings were taken at 450 nm utilized a microplate reader. The EdU staining kit (C0075S, Beyotime, China) was utilized to further evaluate fibroblast proliferation. Cells were seeded in 12-well plates, and the designated treatments were administered for a 24-h period. Then, EdU working solution was added, and the incubation continued for an additional 2 h. After labeling, cells were fixed and permeabilized. EdU Click reaction solution was then applied, and the cells were incubated in darkness for 30 min. DAPI was used for nuclear staining. Images were captured utilizing an Olympus FSX100 microscope.

### Cell migration assay

The migratory capacity of fibroblasts was evaluated utilizing the scratch assay and the transwell assay. For the scratch assay, fibroblasts were seeded in 35-mm cell culture dishes and grown to approximately 90% confluence. A wound gap was created in the cell monolayer utilizing a 200 µL sterile pipette tip. The wound area was rinsed with PBS, and the designated treatments were then applied to the wells. Images of the wounds were captured at 0, 12, and 24 h post-wounding, and the extent of migration was measured utilizing ImageJ software. In the transwell assay, fibroblasts were seeded at a density of 3 × 10^4^ cells/well in the upper chamber of transwell 24-well plates (Corning, USA). Varying concentrations of DPC-Exos were introduced to the upper chamber. 500 µl of basal medium was added to the lower chamber, and the plates were incubated for 24 h. Then, cells that did not migrate through the membrane were carefully removed, and the chamber was fixed with 4% paraformaldehyde. The migrated cells were then stained with 0.5% crystal violet solution (Boster) for 10 min at room temperature. After rinsing with PBS to remove excess dye, the migrated cells were visualized utilizing an Olympus FSX100 microscope.

### Real-time PCR analysis

The expression of each gene was measured utilizing qPCR. Total RNA was extracted from cells and tissue samples utilizing TRIzol Reagent (Takara, Japan) according to the manufacturer’s protocol. Following quality and concentration assessment, 600 ng of RNA was reverse transcribed into cDNA utilizing the Prime Script™ RT reagent Kit (Takara). The resulting cDNA was amplified with SYBR Green PCR Master Mix and gene-specific primers utilizing the CFX Connect system (Bio-Rad), with GAPDH as an internal control. The PCR conditions consisted of an initial denaturation at 95 ℃ for 30 s, and amplified for 40 cycles (95 ℃ for 15 s, 60 ℃ for 30 s, and 70 ℃ for 1 min). Primer sequences are offered in Table 1.

**Table 1:** Primer pairs used for PCR

Gene (mouse)	Primers Sequence (5’-3’)	
CTNNB	Forward	GAGGACAAGCCACAAGATTACA
	Reverse	CCAAGATCAGCAGTCTCATTCC
ALPP	Forward	CCCTGAGTACCCAGATGACTA
	Reverse	AGTGCGGTTCCACACATAC
LEF1	Forward	GGCACCTGTTATCCTACTGAAA
	Reverse	GCTCCATTACGACAGGGATTAG
NOG	Forward	CATGCCGAGCGAGATCAAA
	Reverse	CAGCCACATCTGTAACTTCCTC
GAPDH	Forward	GGTGAAGGTCGGTGTGAACG
	Reverse	CTCGCTCCTGGAAGATGGTG

### Western blot

Protein levels of β-catenin, ALP, Lef1, and Noggin were measured by Western blot analysis. Protein was extracted from cells and tissue samples utilizing lysis buffer (RIPA lysis buffer: protease inhibitor = 1000:1), and protein concentrations were determined utilizing a BCA kit (AR0146, Boster) according to the kit instructions. Protein samples were resolved on 10% SDS-PAGE gels and transferred to PVDF membranes. Membranes were blocked for 2 h at room temperature with 5% non-fat milk. The following primary antibodies were used: β-catenin (8480T, CST, 1:1000), ALP (4060T, CST, 1:2000), Lef1 (ab137872, Abcam, 1:1000), Noggin (30023-1-AP, Proteintech, 1:1000), and GAPDH (GB15004-100, Servicebio, 1:3000). Protein bands were visualized utilizing an ECL Kit (P0018FS, Beyotime). Protein band intensities were quantified utilizing ImageJ, and graphical representations were generated utilizing GraphPad Prism 8.0 software.

### Hair reconstitution assay

Hair patch assays were performed to measure the in vivo hair-inducing capacity of exosome-treated fibroblasts (Exo-Fbs). Epidermal cells were isolated from newborn C57BL/6J mice pups following established protocols [31]. In brief, neonatal mouse skin was digested with 0.25% dispase overnight at 4 °C to separate the epidermal and dermal layers. The epidermis was then minced and subjected to further digestion in 0.25% trypsin-EDTA for 5 min. Digestion was terminated by the addition of serum-containing media, and cell suspensions were filtered through a 100-µm mesh. The resultant filtrate was centrifuged at 300×*g*, and the pelleted cells were resuspended in PBS and enumerated.

For in vivo implantation, 16 nude mice were randomly assigned to four groups: experimental groups consisting of epidermal cells combined with fibroblasts treated with or without DPC-Exos; a positive control group comprised of epidermal cells combined with DPCs; and a control group receiving epidermal cells alone. were used as the positive group, and epidermal cells alone as the control groups. Inducing dermal cells (2 × 10^6^) were combined with epidermal cells (1 × 10^6^) in a total volume of 100 µL PBS and subcutaneously injected into the dorsal region of each nude mouse [13, 32]. Mice were photographed on days 7, 14, and 21 post-transplantation, and tissue samples were collected on day 21 for histopathological analysis.

### Wound healing experiments in mice

To evaluate the effect of DPC-Exos on wound healing, 8-week-old male C57BL/6J mice were randomly assigned to groups (*n* = 5). Full-thickness round excisional wounds (diameter = 1 cm) were created in the dorsal skin of each mouse, and silicone rings were affixed around the wounds to prevent contraction. On days 2, 4, 6, 8, and 10 post-wounding, DPC-Exos (100 µg in 100 µl PBS per wound) or an equal volume of PBS was subcutaneously administered into the dorsal skin surrounding the wounds. Wound images were acquired at various time points (days 0, 3, 7, 10, and 14 post-wounding). The residual wound area of PBS group and DPC-Exos group at different time points were quantified by ImageJ software, and GraphPad Prism 8 software was used to analyze the residual wound area at the same time point between PBS group and DPC-Exos group. The hair coverage area of PBS group and DPC-Exos group was quantified by ImageJ software, then analyzed by GraphPad Prism 8 software to determine the difference of hair growth between PBS group and DPC-Exos group. Mice were sacrificed 14 days post-wounding, and skin samples were harvested for the following histological analysis.

### Histopathology analysis

Collected tissue samples were fixed in 4% paraformaldehyde, dehydrated, embedded, and sectioned at 5 μm thickness. H&E and Masson’s trichrome staining were performed to measure histological changes and collagen deposition, respectively. For immunofluorescence staining, sections were deparaffinized and treated with 3% H_2_O_2_ for 15 min at 37 °C to quench endogenous peroxidase activity. Non-specific binding was then blocked with 5% BSA in PBS for 1 h. Then, slides were incubated overnight at 4 °C with primary antibodies against β-catenin (8480T, CST, 1:200), alkaline phosphatase (ALP; A25629, ABclonal, 1:100), Lef1 (ab137872, Abcam, 1:200), and Noggin (ab16054, Abcam, 1:175). The following day, slides were washed with PBS and incubated for 1 h in the dark with Alexa Fluor 594-conjugated anti-rabbit secondary antibody (Abcam, 1:200). DAPI is used for nuclei staining. Images were acquired utilizing a Pannoramic MIDI scanner (3DHISTECH, Hungary).

### Wnt/β-catenin inhibitor

XAV939 selectively inhibits downstream β-catenin signaling in the Wnt pathway [33]. We employed XAV939(cat. no. HY-15,147, MCE) to inhibit the Wnt pathway in fibroblasts and during wound healing. For in vitro experiments, the inhibitor group was stimulated with 10 µM XAV939, the DPC-Exos + XAV939 group was treated with DPC-Exos (20 µg/mL) + 10µM XAV939, and the control group received an equal volume of DMSO. For the in vivo assay, mice in XAV939 and DPC-Exos + XAV939 groups received four intraperitoneal injections of XAV939 four times at a dose of 1.25 mg/kg each on day 1.

### Animals

C57BL/6J mice(6∼8 weeks old), newborn C57BL/6J mice, and 6-week-old nude mice (Balb/c-nu) were all obtained from the Experimental Animal Center of Air Force Medical University (Xi’an, China). All animal experiments were conducted in strict accordance with guidelines established by the Experimental Animal Committee of Air Force Medical University.

### Statistical analysis

All data were analyzed utilizing GraphPad Prism 8 software and are presented as mean ± standard deviation. A *t*-test was used for comparisons between two groups, and one-way ANOVA was used for multi-group comparisons. *P* < 0.05 was considered statistically significant.

## Results

### Characterization of DPCs and DPC-Exos

**Fig. 1:**
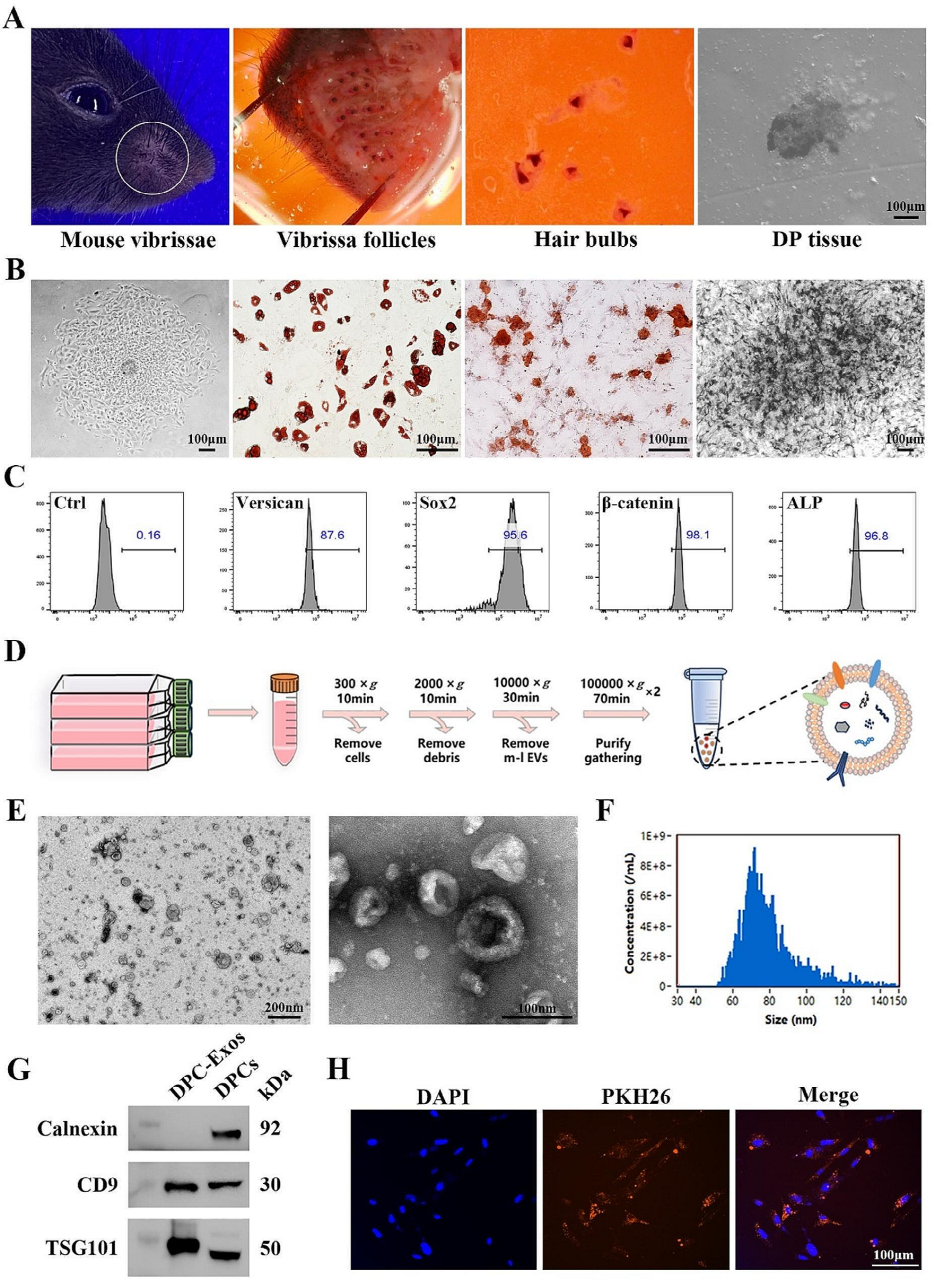
Isolation and identification of DPCs and DPC-Exos. **A** The process of separating DP tissues from mouse vibrissa follicles through microdissection coupled with collagenase digestion. **B** The growth, adipogenic differentiation, osteogenic differentiation, and alkaline phosphatase staining of DPCs, scale bar = 100 μm. **C** Flow cytometry analysis of DPCs, the positive expression of DPC markers (Versican 87.6%, Sox2 95.6%, β-catenin 98.1%, ALP 96.8%). **D** Schematic diagram of the exosome extraction process. **E** The morphology of DPC-Exos analyzed by TEM (nm). **F** The particle size distribution of DPC-Exos measured by NTA. **G** Immunoblot analysis of known exosome markers (CD9, TSG101) and negative markers (Calnexin). **H** Representative images of the internalization of PKH26-labeled DPC-Exos into fibroblasts (Red: PKH26 labeled DPC-Exos, Blue: DAPI). Scale bar = 100 μm

DP tissues were successfully separated through microdissection combined with collagenase digestion. After approximately 30 min of digestion in 0.2% type I collagenase, the hair bulb structure loosened, and DP tissue gradually dissociated from the surrounding tissues. The dissociated DP tissue exhibited a conical or round shape with high density (Fig. 1A). Primary DPCs migrated from the DP tissue in 3 days of seeding, and by day 7, the DPCs had almost completely migrated out of the DP tissue. Microscopic study indicated that adherent DPCs displayed a short, spindle-like morphology and exhibited aggregative behavior during cell culture. Oil Red O and Alizarin Red S staining demonstrated the presence of lipid droplets and calcium deposition in DPCs following adipogenic and osteogenic differentiation-induced culture, respectively. In addition, DPCs exhibited strong positive Alkaline phosphatase (ALP) activity (Fig. 1B). Versican, β-catenin, and ALP are recognized as molecular markers of DPCs. Sox2 is known to be expressed in DPCs and significantly regulates hair pigmentation [34]. Flow cytometric analysis indicated that the isolated DPCs were highly positive for DPC-specific markers [35] (Fig. 1C). Collectively, these findings confirmed that the isolated cells exhibited characteristics consistent with typical DPCs.

The protocol employed for DPC-Exos separation is depicted in Fig. 1D. TEM analysis indicated that DPC-Exos have a cup- or sphere-shaped morphology. NTA analysis demonstrated that the particle diameters of DPC-Exos ranged from 50 to 150 nm, with an average size of 79.0 nm (Fig. 1E, F). Western blot analysis further confirmed that DPC-Exos were positive for established exosomal markers CD9 and TSG101, while being negative for the cellular marker Calnexin (Fig. 1G). The results obtained from TEM, NTA, and Western blot analysis collectively indicated the successful isolation of DPC-Exos.

### DPC-Exos promoted the proliferation and migration of fibroblasts in a concentration-dependent manner

**Fig. 2:**
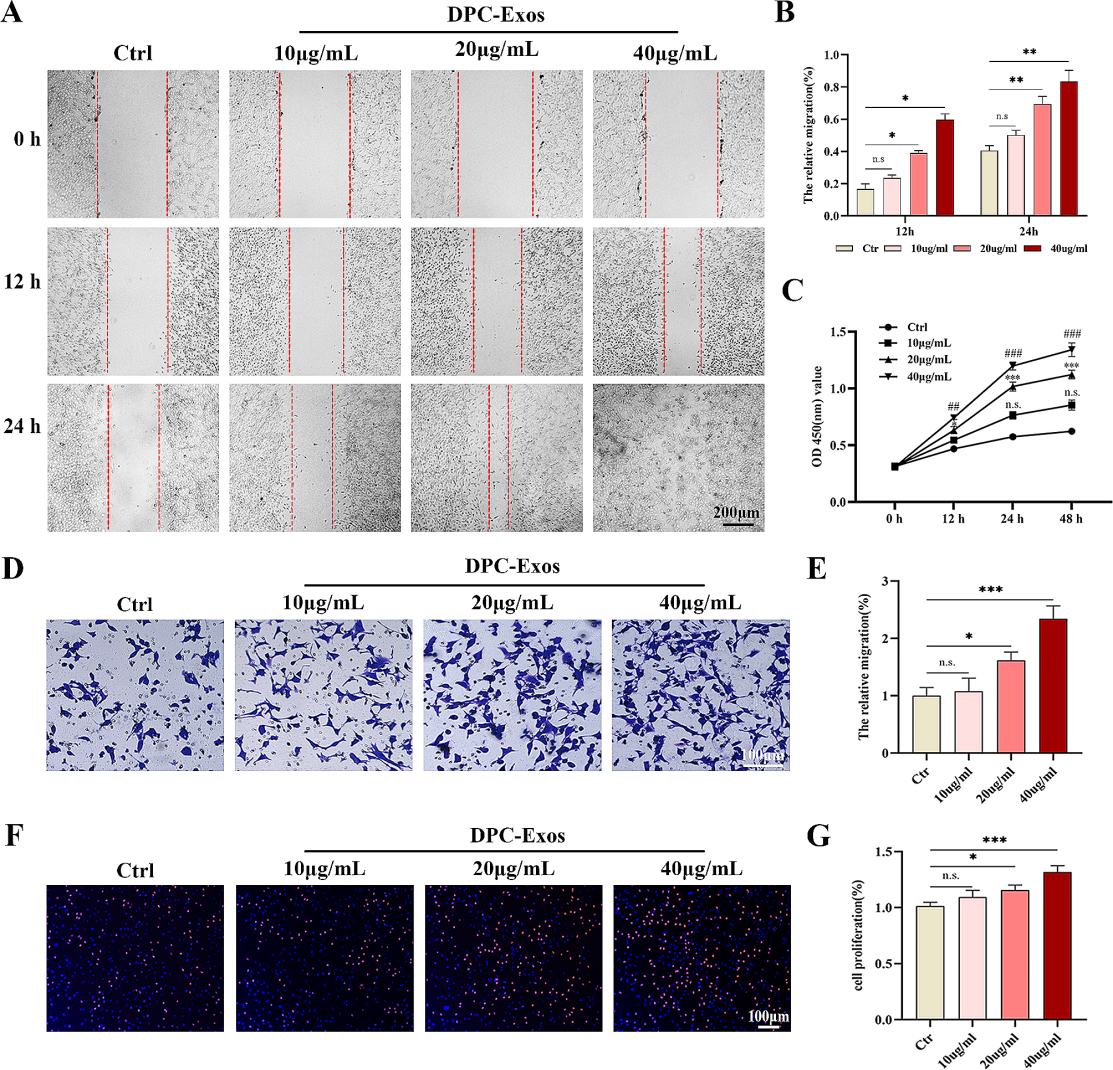
DPC-Exos promoted the proliferation and migration of fibroblasts in a concentration-dependent manner. **A** Scratch assay detected the migration of fibroblasts after different concentrations of DPC-Exos treatment, scale bar = 200 μm. **B** Statistical analysis of scratch assay. **C** The proliferative activity of fibroblasts after different concentrations of DPC-Exos treatment was assessed by CCK-8 assay. **D** The migration of fibroblasts after different concentrations of DPC-Exos treatment was measured by transwell assay, scale bar = 100 μm. **E** Statistical analysis of transwell assay. **F** EdU staining detected the proliferative activity of fibroblasts after DPC-Exos treatment, scale bar = 100 μm. G Statistical analysis of EdU staining. n.s.: no significant difference, ^*^P < 0.05, ^**^P < 0.01, ^***^P < 0.001, ^##^*P* < 0.01, and ^###^*P* < 0.001

Fibroblasts constitute the primary cell population of the dermis and are widely recognized as crucial factors of wound healing outcomes [36]. While DPC-Exos have demonstrated the ability to enhance the proliferation and migration of hair follicle stem cells and hair matrix cells, their effect on fibroblasts remains to be explained. To evaluate the effects of DPC-Exos on fibroblasts, we first assessed their uptake by these cells. Co-incubation of PKH26-labeled DPC-Exos with fibroblasts for 24 h indicated the internalization of labeled DPC-Exos by fibroblasts (Fig. 1H). To further appraise the effect of DPC-Exos on fibroblasts, we treated them with varying concentrations of DPC-Exos. CCK-8 assay results indicated that DPC-Exos significantly accelerated fibroblast proliferation in a concentration-dependent fashion compared to the control group. A statistically significant difference was observed at a DPC-Exos concentration of 20 µg/mL, with the most rapid proliferation occurring at 24 h of co-incubation (Fig. 2C). EdU staining corroborated the stimulatory effect of DPC-Exos on fibroblast proliferation (Fig. 2F). Moreover, scratch assay and transwell assay findings indicated that DPC-Exos enhanced fibroblast migration in a concentration-dependent manner. Specifically, exosome concentrations of 20 µg/mL and 40 µg/mL significantly enhanced fibroblast migration rates compared to the control group (Fig. 2A, E). These results collectively demonstrate that DPC-Exos promote fibroblast proliferation and migration, establishing a basis for their potential role in accelerating wound healing through fibroblast regulation.

### DPC-Exos promoted the expression level of molecules associated with hair-inducing capacity in fibroblasts

**Fig. 3:**
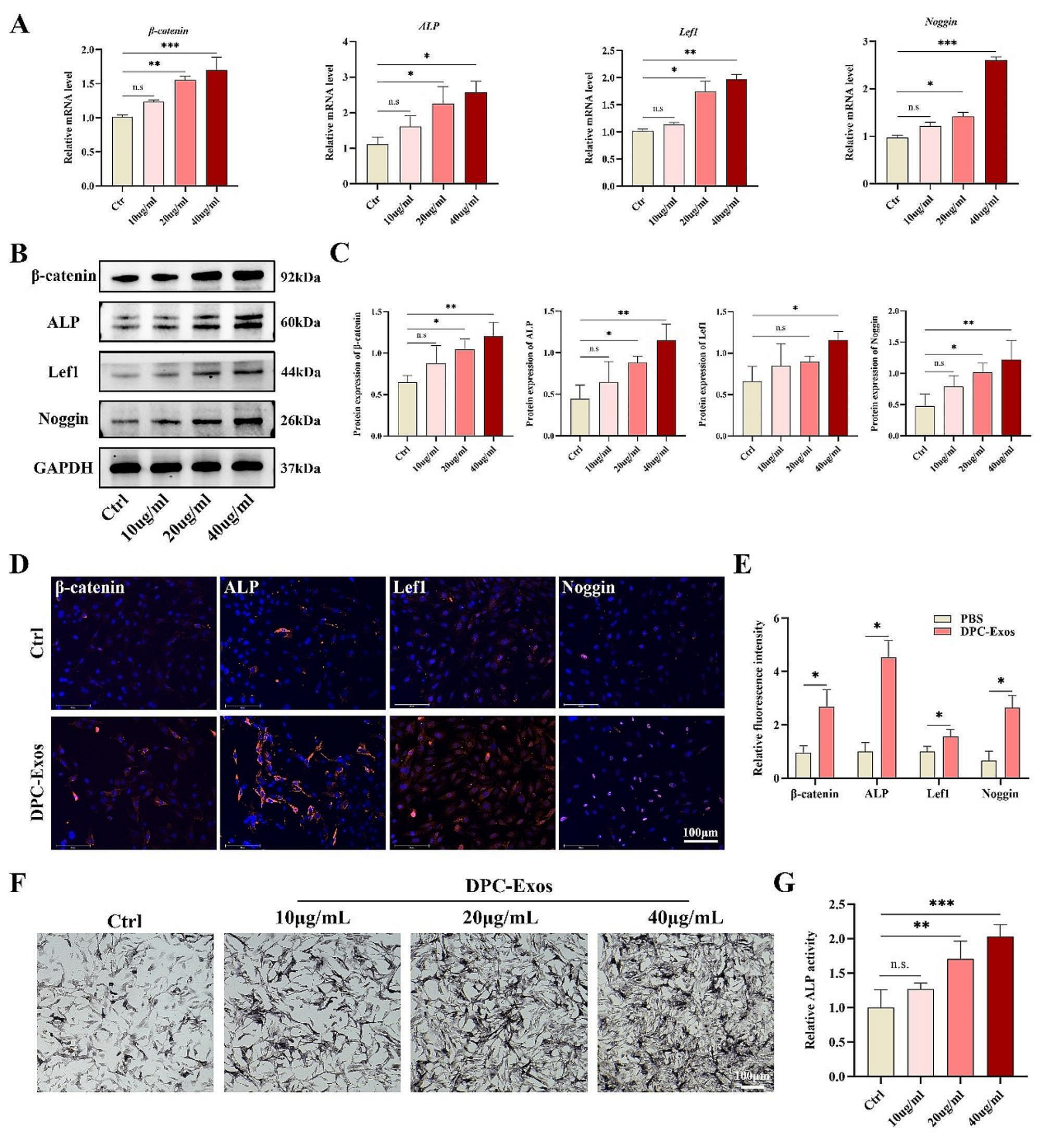
DPC-Exos enhanced the expression of β-catenin, ALP, Lef1, and Noggin in fibroblasts. **A** Fibroblasts were treated with different concentrations of DPC-Exos. The gene expression of β-catenin, ALP, Lef1, and Noggin in fibroblasts was detected by qPCR. **B**, **C** Western blot and quantitative analysis showed that the protein expression of β-catenin, ALP, Lef1, and Noggin in fibroblasts was improved after DPC-Exos treatment. **D**, **E** Immunofluorescent staining and quantitative analysis of β-catenin, ALP, Lef1, and Noggin in fibroblasts after PBS (Ctrl) and DPC-Exos (20 µg/mL) treatment, Blue: DAPI, Red: Target molecule, scale bar = 125 μm. **F** ALP staining of fibroblasts after different concentrations of DPC-Exos treatment, scale bar = 100 μm. **G** Statistical analysis of ALP staining assay. The data was shown as mean ± SD. n.s., no significant difference; ^*^P < 0.05, ^**^P < 0.01, and ^***^P < 0.001

β-catenin and ALP are widely recognized markers of DPCs and are associated with the hair-inducing activity of cells [37, 38]. Noggin is expressed by DPCs and is indicative of the onset of hair growth [39]. Lef1 is a DPC marker, and Lef1^+^ fibroblasts represent a unique subset of cells that can support HF regeneration [11]. To analyze the hair-inductive potential of fibroblasts following DPC-Exos treatment, we employed qPCR and Western blot to determine the expression levels of β-catenin, ALP, Lef1, and Noggin in fibroblasts. Our findings demonstrated that, compared to the control group, DPC-Exos at concentrations of 20 µg/ mL and 40 µg/ mL significantly upregulated the mRNA and protein expression of β-catenin, ALP, Lef1, and Noggin in fibroblasts (Fig. 3A, B). Immunofluorescence staining further corroborated this effect of DPC-Exos (Fig. 3D). Moreover, ALP staining indicated that DPC-Exos enhanced ALP activity in fibroblasts (Fig. 3F). Collectively, these results demonstrate that DPC-Exos can promote the expression of molecules associated with hair-inducing capacity in fibroblasts. We propose that DPC-Exos may facilitate the acquisition of DPC characteristics by fibroblasts, finally leading to their transformation into DPCs.

### Hair reconstitution assay indicated that Exo-Fbs could induce HF neogenesis when combined with neonatal mice epidermal cells

**Fig. 4:**
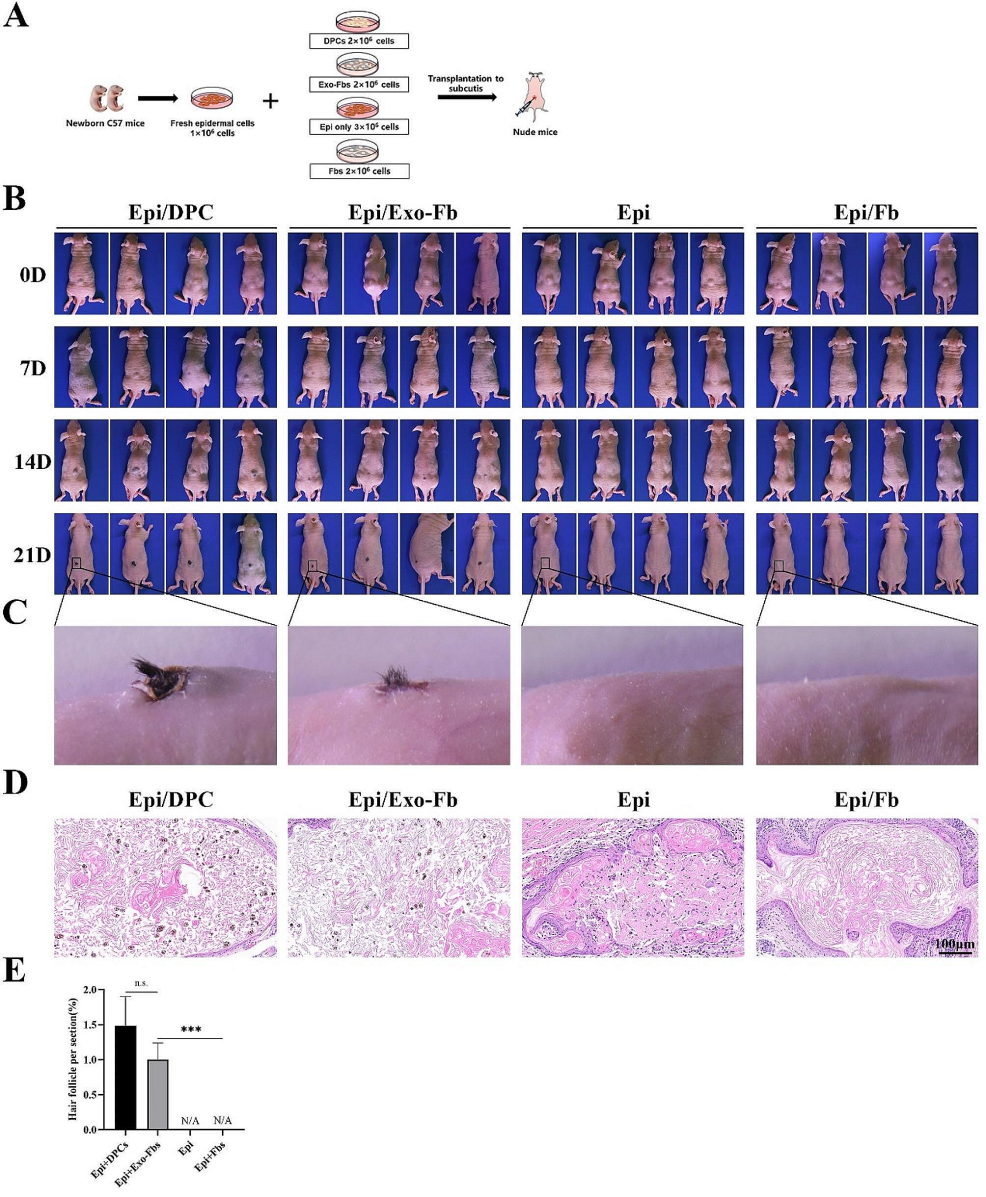
In vivo hair reconstitution assay. **A** The protocol of hair reconstruction assay. Four groups of nude mice were subcutaneously injected with Epi + DPCs, Epi + Exo-Fbs, Epi alone, and Epi + Fbs, respectively. **B** Mice were imaged at day 0, 7, 14, and 21 after cell implantation to observe the new hair growth in different groups. **C** Stereo images of new hair based on transplantation of different combinations of cells. **D** H&E staining of transplant sites from different treatment groups, scale bar = 100 μm. **E** The histogram showed the relative number of newly formed HFs in different groups. Epi: neonatal mice epidermal cells; Fbs: fibroblasts; Exo-Fbs: DPC-Exos treated fibroblasts. n.s., no significant difference, ^***^P < 0.001

Hair reconstitution assay was conducted to further evaluate the in vivo hair-inducing capacity of Exo-Fbs. As depicted in Fig. 4B, at 7 days post-implantation, obvious black hair clumps were observed beneath the skin at the injection site in both the Epi/DPCs and Epi/Exo-Fbs group. However, no obvious black bulge was detected in the Epi or Epi/Fbs groups. Two weeks post-implantation, the hair clumps were larger and clearer beneath the skin of the nude mice in both the Epi/DPCs and Epi/Exo-Fbs groups. Due to the variable orientation of newly formed HFs, hair tufts were unable to penetrate the skin surface. To facilitate outward hair growth, a portion of the skin at the transplantation sites was carefully removed. Three weeks post-implantation, abundant blank hairs were apparent in the Epi/DPCs group, while new hair formation was observed in the Epi/Exo-Fbs group, albeit to a lesser extent than the Epi/DPCs group. Specifically, neither the epidermal alone nor the epidermal combined with untreated fibroblasts exhibited evident new hair development (Fig. 4C). Moreover, H&E staining of nude mice tissues indicated that the DPCs and Exo-Fbs induced the formation of concentric circle-like structures composed of epidermal cells, with scattered new HFs within. Although the untreated fibroblasts also induced concentric circular layered structures, no obvious follicle-like structure were formed (Fig. 4D). Hair-inducing capacity of dermal cells determined by comparing the average number of new HFs in different groups. The DPCs were most effective at inducing new hair formation, and Exo-Fbs also induced more neogenic hairs than untreated fibroblasts. These findings from the hair reconstitution assay offer evidence for the hair-inducing capacity of Exo-Fbs in vivo.

### DPC-Exos accelerated wound healing and promoted HF regeneration in the skin full-thickness excision wounds of C57BL/6J mice

**Fig. 5:**
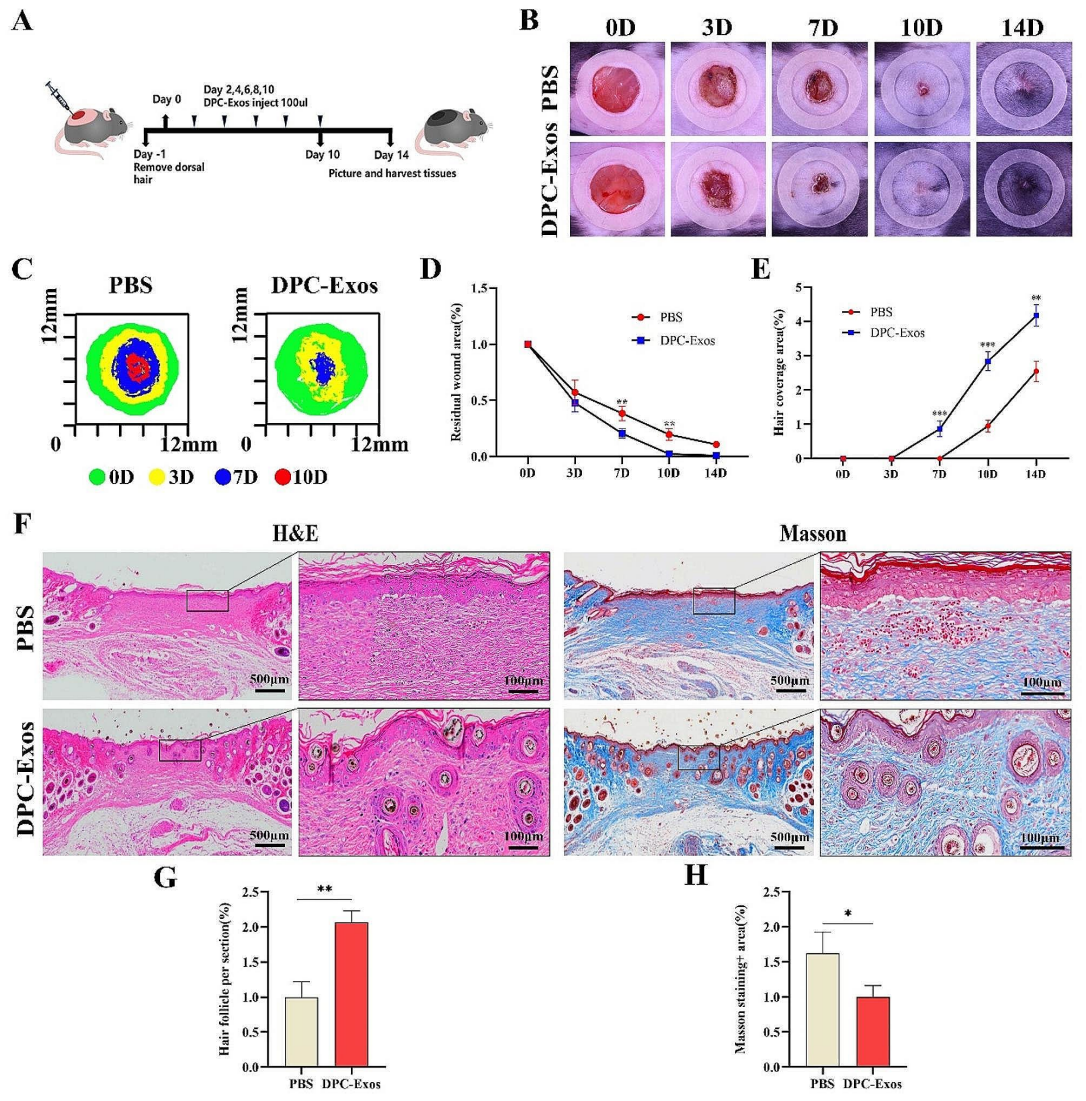
DPC-Exos accelerated re-epithelialization and promoted HF regeneration in full-thickness skin excisional wound of mice. **A** Schematic diagram of animal experiment procedures. **B** Digital photographs of wound area treated with PBS or DPC-Exos at day 0, 3, 7, 10, and 14 post-wounding. **C** Traces of wound-bed closure during 14 days between PBS group and DPC-Exos group. **D** Line chart showed the change of residual wound area over time between PBS group and DPC-Exos group. **E** Line chart showed the hair growth changes between PBS group and DPC-Exos group. **F** H&E and Masson staining of tissue samples from PBS group and DPC-Exos group 14 days post-wounding, scale bar = 500 μm, 100 μm. **G**, **H** Statistical analysis of the number of new HFs and Masson staining^+^ area between PBS group and DPC-Exos group. The data was shown as mean ± SD. ^*^P < 0.05, ^**^P < 0.01, and ^***^P < 0.001

Given the potent effect of DPC-Exos on promoting HF development, we sought to analyze their effect on wound healing in a murine model of full-thickness skin excision. Wound healing assays indicated a significantly accelerated rate of closure in the DPC-Exos group compared to the PBS control group. This was evidenced by smaller wound areas on days 7 and 10 post-wounding, with statistically significant differences observed between the groups (*P* < 0.05) (Fig. 5B-D). In addition, the DPC-Exos group exhibited earlier and more rapid hair growth (starting on the 7th-day post-wounding). The hair coverage area in the DPC-Exos group was significantly greater than that of the PBS group at days 7, 10, and 14 post-wounding (Fig. 5B, E).

**Fig. 6:**
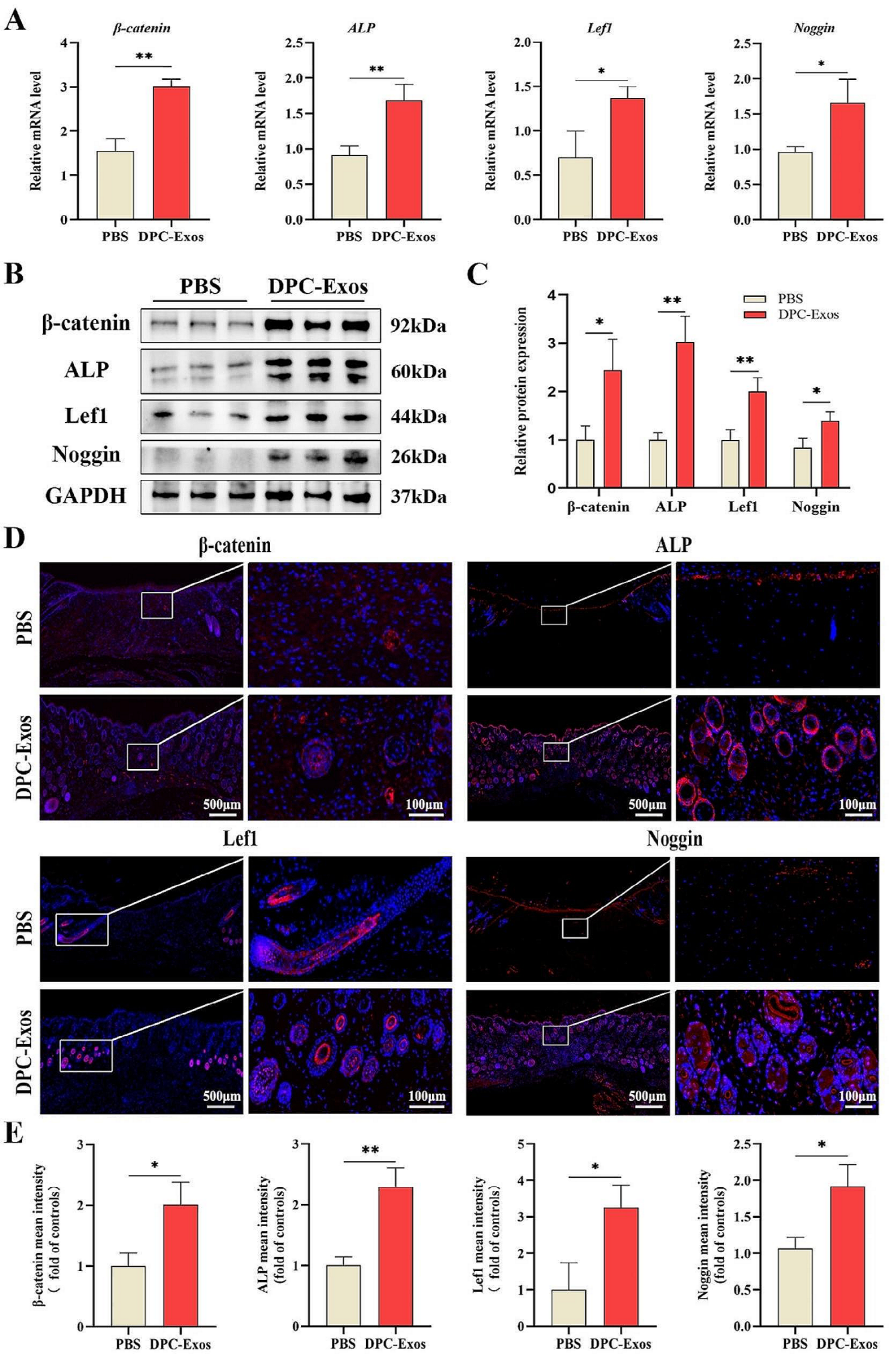
DPC-Exos promoted the expression of β-catenin, ALP, Lef1, and Noggin in tissues. **A** Gene expression of β-catenin, ALP, Lef1, and Noggin in tissue samples was detected by qPCR. **B** Western blot was performed to detect the protein expression levels of β-catenin, ALP, Lef1, and Noggin in PBS and DPC-Exos treated mice. **C** Quantitative analysis of Western blot. **D** Immunofluorescent staining of β-catenin, ALP, Lef1, and Noggin in tissue samples from PBS group and DPC-Exos group. Blue: DAPI; Red: Target molecules, scale bar = 500 μm, 100 μm. **E** Quantitative analysis of the mean fluorescent intensity of β-catenin, ALP, Lef1, and Noggin in PBS and DPC-Exos treated mice. The data was shown as mean ± SD. ^*^P < 0.05, ^**^P < 0.01

Histological analysis utilizing H&E and Masson staining of tissue samples indicated the formation of new HFs at the wound site in the DPC-Exos group; whereas, collagen deposition was reduced in this group. We hypothesize that this HF regeneration may be linked to the hair-inducing activity of fibroblasts. Besides, qPCR and Western blot analyses of tissue samples demonstrated significantly enhanced expression levels of β-catenin, ALP, Lef1, and Noggin in the DPC-Exos group compared to the PBS group (Fig. 6A, B). These findings were further corroborated by tissue immunofluorescence staining, which confirmed the upregulation of β-catenin, ALP, Lef1, and Noggin in the dermis and new hair follicles (Fig. 6D, E). Accordingly, our wound healing assays demonstrate that DPC-Exos can accelerate wound closure, promote HF regeneration, and enhance the expression of molecules associated with hair inductive activity in tissues.

### DPC‑Exos activated Wnt/β-catenin signaling pathway in fibroblasts

**Fig. 7:**
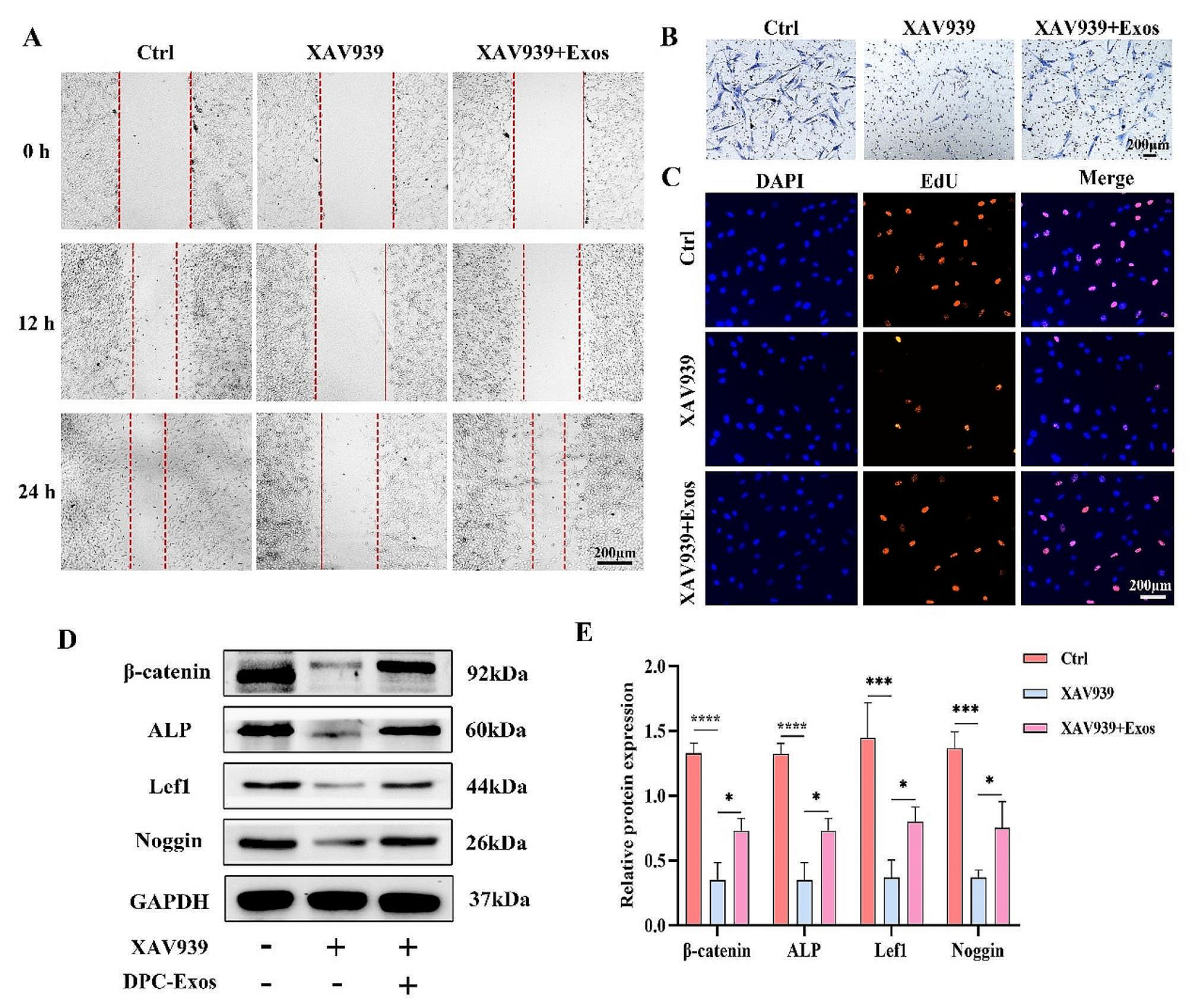
Effect of DPC-Exos on fibroblasts after application of Wnt inhibitor. **A**, **B** Scratch and transwell assay showed that XAV939 hindered the migration of fibroblasts, and DPC-Exos partially rescued the inhibitory effect of XAV939 on fibroblasts, scale bar = 200 μm. **C** EdU staining showed that XAV939 inhibited the proliferation of fibroblasts, and DPC-Exos partially rescued the inhibitory effect of XAV939 on fibroblasts, scale bar = 200 μm. **D** Western blot was conducted to detect the expression levels of β-catenin, ALP, Lef1, and Noggin in fibroblasts in the control, XAV939, and XAV939 + exosomes groups. **E** Quantitative analysis of Western blot. XAV939 inhibited the expression of molecules related to hair-inducing activity in fibroblasts, and DPC-Exos could partially reverse the inhibition of XAV939 by promoting the expression of β-catenin, Lef1 and activating the Wnt pathway. The data was shown as mean ± SD. ^*^P < 0.05, ^**^P < 0.01, ^***^P < 0.001, and ^****^P < 0.0001

The upregulation of β-catenin and Lef1 indicated the activation of the Wnt/β-catenin signaling pathway by DPC-Exos. Wnt inhibitor XAV939 was utilized to verify the effect of DPC-Exos on the Wnt/β-catenin pathway. Here, fibroblasts were treated with DMSO, XAV939, and XAV939 + Exos. The results of the scratch assay, transwell assay, and EdU staining collectively demonstrated that XAV939 significantly inhibited the proliferation and migration of fibroblasts, suggesting that blocking the Wnt/β-catenin signaling pathway could hinder the activity of fibroblasts. This observation aligns with previous findings regarding the inhibition of the Wnt/β-catenin signaling pathway in fibroblasts [15]. Specifically, in the XAV939 + Exos group, the proliferative and migratory activity of fibroblasts was enhanced compared to the XAV939 group (Fig. 7A-C), indicating that DPC-Exos reduced the inhibitory effect of XAV939 on fibroblasts. In addition, Western blot analysis indicated that the protein expression levels of β-catenin, ALP, Lef1, and Noggin in fibroblasts were significantly reduced following XAV939 treatment. However, DPC-Exos attenuated the inhibitory effect of XAV939, leading to an increase in the expression levels of these molecules (Fig. 7D, E). Accordingly, these results suggested that DPC-Exos could promote fibroblast activities through the activation of the Wnt/β-catenin signaling pathway.

### Promoting effects of DPC-Exos on wound healing was inhibited by XAV939

**Fig. 8:**
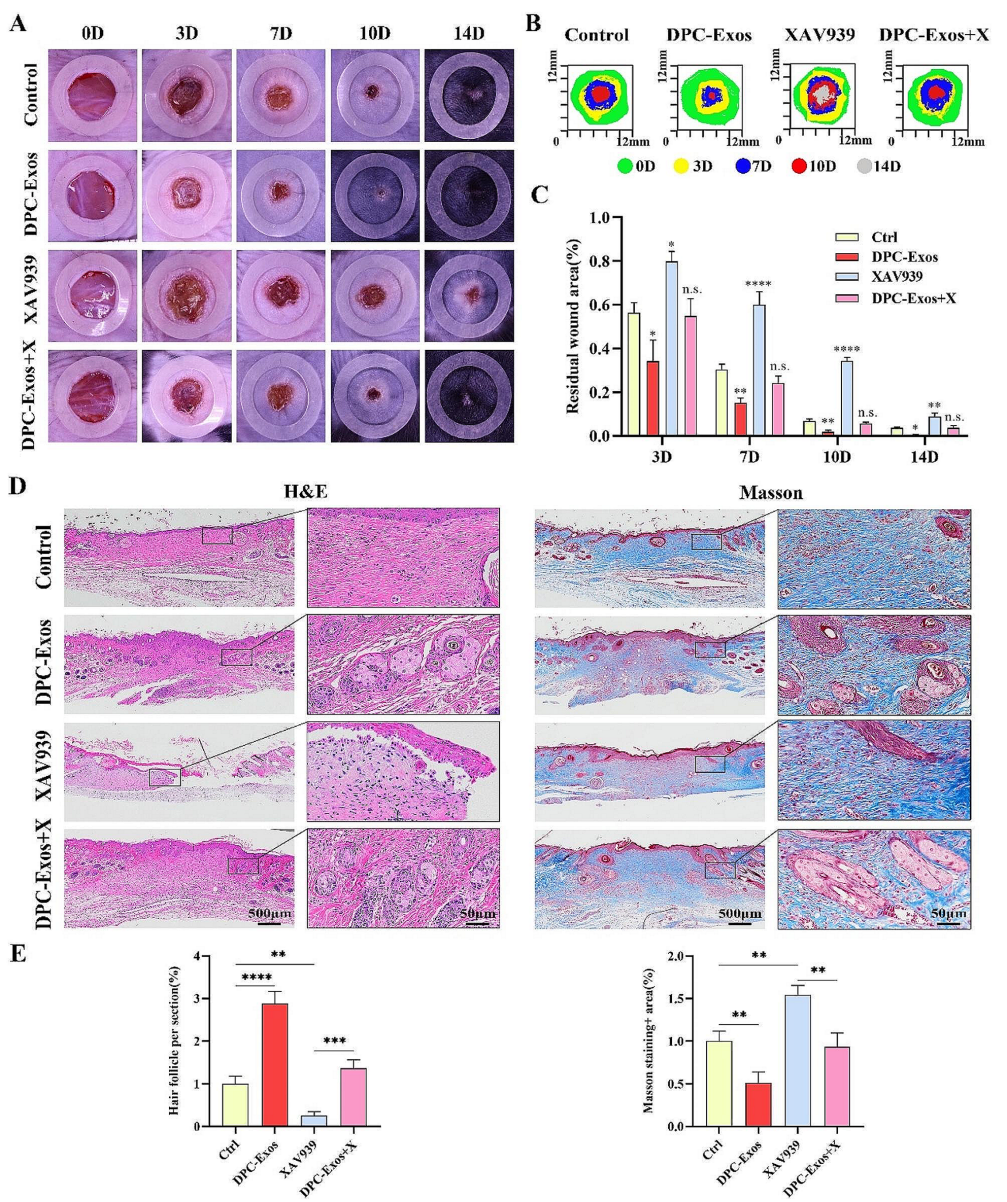
Effects of DPC-Exos on wound healing after application of Wnt pathway inhibitor. **A** Digital photos of wound area in different groups at day 0, 3, 7, 10, and 14 post-wounding. **B** Traces of wound-bed closure during 14 days for each treatment category. **C** Histogram showed the relative change of wound area during 14 days in control, DPC-Exos, XAV939, and DPC-Exos + X groups. DPC-Exos accelerated wound healing and HF regeneration, and the promotion effect was reduced after XAV939 treatment. **D** H&E and Masson staining of tissue samples from control, DPC-Exos, XAV939, and XAV939 + Exos groups on day 14 post-wounding, scale bar = 500 μm, 50 μm. **E** Statistical analysis of relative hair follicle number and Masson staining^+^ area in different treatment groups. The data was shown as mean ± SD. n.s., no significant difference; ^*^P < 0.05, ^**^P < 0.01, ^***^P < 0.001, and ^****^P < 0.0001

To further verify whether DPC-Exos regulates HF regeneration by activating the Wnt/β-catenin signaling pathway during wound healing, we employed XAV939 to block the Wnt pathway in mice. As depicted in Fig. 8A-C, after treatment with XAV939, wound closure was significantly slower compared to other groups, suggesting that blocking the Wnt/β-catenin signaling pathway could impede wound healing. Specifically, the wound closure rate and the number of new HFs in DPC-Exos + X group was significantly reduced compared to the DPC-Exos group, which suggested that blocking the Wnt/β-catenin signaling pathway could inhibit the promoting effects of DPC-Exos on wound healing and HF regeneration. H&E and Masson staining indicated the formation of new HFs and sebaceous glands in the DPC-Exos and DPC-Exos + X groups (Fig. 8D), while the tissue structure was disordered and no HFs formed in the XAV939 group.

**Fig. 9:**
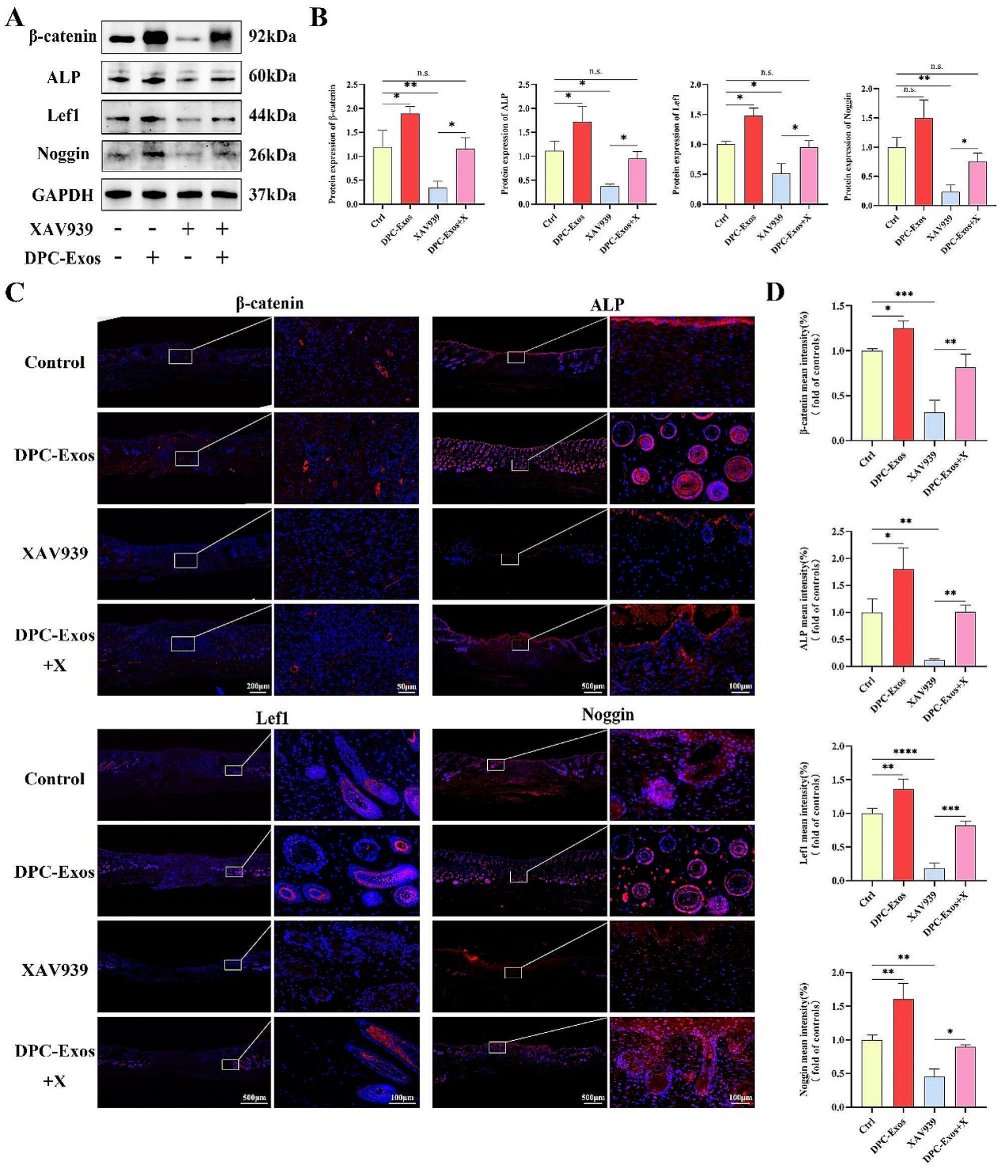
Expression levels of β-catenin, ALP, Lef1, and Noggin in tissue after the use of Wnt pathway inhibitor. **A** Western blot was conducted to detect the protein expression levels of β-catenin, ALP, Lef1, and Noggin in tissue samples of control, DPC-Exos, XAV939, and XAV939 + Exos groups. B Quantitative analysis of Western blot. **C** Immunofluorescent staining of β-catenin, ALP, Lef1, and Noggin in tissue samples of control, DPC-Exos, XAV939, and XAV939 + Exos groups 14 days post-wounding after the use of Wnt pathway inhibitor. Blue: DAPI; Red: Target molecules, scale bar = 500 μm, 100 μm. **D** Quantitative analysis of the mean fluorescent intensity of β-catenin, ALP, Lef1, and Noggin in tissue samples from different treatment group. The data was shown as mean ± SD. n.s., no significant difference; ^*^P < 0.05, ^**^P < 0.01, ^***^P < 0.001, and ^****^P < 0.0001

Western blot and quantitative analyses indicated that compared to the control group, DPC-Exos upregulated the expression of β-catenin, ALP, Lef1 and Noggin in tissues, which was consistent with the results of our previous animal experiment. In addition, XAV939 significantly reduced the expression levels of β-catenin, ALP, Lef1, and Noggin in tissues, whereas, DPC-Exos treatment led to an increase in the expression of these molecules. No significant difference in the expression levels of β-catenin, ALP, Lef1, and Noggin was observed between the control group and the DPC-Exos + X group (Fig. 9A, B). In addition, immunofluorescence staining demonstrated that XAV939 treatment significantly reduced the fluorescence intensity of β-catenin, ALP, Lef1, and Noggin in the dermis. In contrast, DPC-Exos treatment increased the fluorescence intensity of these molecules (Fig. 9C, D). These findings further confirm that DPC-Exos can promote HF regeneration during wound healing by activating Wnt/β-catenin signaling pathway.

## Discussion

Deep skin injuries heal with scar formation, which prevents the regeneration of skin appendages such as HF and glands. HF regeneration during wound healing is essential for restoring skin structure and function, representing an optimal healing approach and a hallmark of regenerative wound healing [40, 41]. Previously, it was believed that HF morphogenesis primarily occurred during embryonic development, and that HF loss in adult mammals due to deep skin injuries was irreparable. However, in 2007, Ito et al. [42] observed functional HF regeneration in large skin wounds (> 1 cm^2^) in adult mice, paving the way for new analyses into HF regeneration during wound healing. Although DPC-Exos has been extensively studied in promoting HF regeneration, its role and mechanism in wound healing remain unclear.

Wound healing is a complex biological process, that involves a diverse array of cells. Among these, fibroblasts constitute the primary cell population that dictates wound healing outcomes. Specifically, Li et al. demonstrated that exosomes derived from adipose stem cells can enhance fibroblast proliferation and expedite wound healing through the activation of the Wnt/β-catenin signaling pathway [15]. In this study, we observed that DPC-Exos similar to exosomes derived from other stem cells, could enhance the proliferation and migration of fibroblasts, which undoubtedly contributing to wound healing. Considering the hair inductive properties of DPCs and their homology with fibroblasts, numerous efforts have been carried out to facilitate the transformation of fibroblasts into DPCs. These strategies consist of biomimetic hydrogel encapsulation culture [13], small molecule compounds inducing [12], and suspension culture with FGF2, PDGF and BIO [43]. While these methodologies enhance the hair-inducing capabilities of fibroblasts, they are not conducive to wound healing applications. DPC-Exos, functioning as a natural dermal signaling delivery system, have the capacity to stimulate both the proliferation and differentiation in hair follicle stem cells and hair matrix cells, thereby augmenting the hair-inductive potential of dermal papilla spheres [44, 45]. Through co-incubation of fibroblasts with DPC-Exos, we detected increased expression levels of molecules implicated in the hair-inducing capacity of DPCs (β-catenin, ALP, Lef1, and Noggin) in fibroblasts. Hair reconstitution assay demonstrated that fibroblasts acquired sufficient hair-inducing activity after DPC-Exos treatment, thereby enhanced EMI when interacting with epidermal cells and induced HF neogenesis. These findings collectively suggest that DPC-Exos confer the potential of fibroblasts to transform to DPCs, establishing a foundation for DPC-Exos to facilitate HF regeneration during wound healing.

In the wound healing assay, by monitoring wound bed area and hair growth, we observed that DPC-Exos facilitated wound re-epithelialization. This finding aligns with the established role of mesenchymal stem cell-derived exosomes in promoting wound healing. In addition, the DPC-Exos group exhibited significantly increased HF neogenesis and reduced collagen deposition. We hypothesized that DPC-Exos enhanced HF regeneration by modulating fibroblast phenotypes. To explore this mechanism, we analyzed the expression levels of molecules associated with the hair-inducing capacity of DPCs. Gene and protein expression levels of β-catenin, ALP, Lef1, and Noggin were upregulated in the DPC-Exos group. Consistent with these observations, tissue immunofluorescence staining showed that the fluorescence intensity of β-catenin was significantly increased in the dermal layer and newly formed HFs. Lef1, a marker of neogenic HF, also displayed higher fluorescence intensity in the DPC-Exos group. These findings support our hypothesis that DPC-Exos can promote HF regeneration during wound healing by enhancing the hair-inducing activity of fibroblasts.

The Wnt/β-catenin signaling pathway is crucial for HF regeneration [46–48], and a growing body of evidence suggests its activation can promote wound healing [49, 50]. During HF morphogenesis, the dermis offers the ‘first dermal signal,’ which initiates the formation of epidermal placodes. Sustained β-catenin activity in the dermis accelerates HF differentiation and growth [49, 51]. Kazi et al. demonstrated that DPC-Exos, acting as an inducible dermal signal, can enhance hair inductive gene expression in adipose stem cells by enhancing β-catenin activity [52]. In this study, we observed that DPC-Exos elevates the expression of both β-catenin, a crucial Wnt/β-catenin pathway transcription factor, and Lef1, a Wnt effector molecule, in fibroblasts. This observation led us to hypothesize that the effects of DPC-Exos on fibroblasts are mediated through the activation of the Wnt/β-catenin signaling pathway. To study this hypothesis, we employed the Wnt inhibitor XAV939 to specifically inhibit the Wnt pathway in fibroblasts, thereby reducing cellular β-catenin production. The results of in vitro experiments indicated that XAV939 suppressed fibroblast proliferation and migration, however, DPC-Exos ws found to reduce the inhibitory effect of XAV939 by promoting β-catenin production and expression. Moreover, XAV939 significantly reduced protein expression levels of β-catenin, ALP, Lef1, and Noggin in fibroblasts. Specifically, DPC-Exos treatment was able to partially restore the expression of these molecules. Accordingly, these findings indicate that DPC-Exos can enhance the hair-inducing activity of fibroblasts by activating the Wnt/β-catenin signaling pathway.

For the role of the Wnt/β-catenin signaling pathway in wound healing, some studies have demonstrated that activation of the Wnt/β-catenin pathway in fibroblasts results in excessive collagen deposition and scarring [53, 54]. These findings have prompted us to evaluate the role of the Wnt/β-catenin pathway in wound healing. It is well established that in addition to producing and maintaining the extracellular matrix, fibroblasts also critically affect HF regeneration. Phan et al. have demonstrated that Lef1^+^ papillary fibroblasts can regenerate new HFs through interactions with adjacent epidermal cells [11]. Mascharak et al. reported that inhibiting YAP signaling in fibroblasts promotes regenerative healing by activating the Wnt signaling pathway [40]. The Wnt/β-catenin signaling pathway is implicated in multiple stages of wound healing, and inhibiting its activity may affect cell viability and the wound healing process. Moreover, activation of Wnt/β-catenin signaling is essential for HF morphogenesis and maturation. We observed that inhibiting the Wnt pathway also impaired wound re-epithelialization and HF regeneration. While some wounds in the XAV939 group exhibited re-epithelialization, HF regeneration was not observed. In addition, compared to the DPC-Exos group, blocking the Wnt pathway attenuated the stimulatory effect of DPC-Exos on HF regeneration. Besides, the stimulatory effect of DPC-Exos on the expression of β-catenin, ALP, Lef1, and Noggin was also reduced by XAV939. These results suggest that DPC-Exos promotes HF regeneration in wound healing by activating the Wnt/β-catenin signaling pathway.

Fibroblasts are highly dynamic cells in wound healing, and their heterogeneity and plasticity offer opportunities to modulate healing outcomes. Numerous studies have demonstrated the attenuation of fibrotic repair by inhibiting the fibroblast-to-myofibroblast transition and disrupting YAP mechanotransduction in fibroblasts [55, 56]. However, these regulatory approaches may affect fibroblast activity, thereby influencing the overall wound healing process. Some research suggests that regenerative and fibrotic processes are not mutually exclusive during wound healing, and cells with pro-regenerative properties are also present in scars. However, in the absence of intervention, the pro-fibrotic program dominates, finally leading to scar formation. In addition, the inability to regenerate skin appendages during wound healing appears to be linked to the absence of dermal regeneration signals rather than a lack of cellular regenerative capacity [40, 57]. In this study, we introduced DPC-Exos, a hair-inducing dermal signal, into the wound microenvironment and observed its positive effects in promoting HF regeneration during skin injury repair. With advancements in nanobiotechnology, there is increasing interest in the role and applications of exosomes in disease and tissue repair [58]. Chen et al. [24] encapsulated DPC-Exos in a hydrogel material to enhance their stability and enable sustained release, thus prolonging their preservation and functional duration. Besides, several studies have indicated that miRNA [59, 60] derived from DPC-Exos exerts a positive effect on HF regeneration. These findings offer a theoretical foundation and inspiration for the transformation and engineering of DPC-Exos, further establishing the basis for their clinical application. Our study demonstrates that DPC-Exos can promote HF regeneration during wound healing. However, this alone is insufficient for achieving complete regenerative wound healing, which also necessitates the re-establishment of normal matrix ultrastructure and the restoration of mechanical robustness. Moreover, the contents of DPC-Exos, which are crucial for their function, require further research. In future studies, we will conduct more in-depth and explorations of these aspects to achieve superior regenerative wound healing.

## Conclusion

In conclusion, this study has demonstrated that DPC-Exos have the capability to stimulate the proliferation and migration of fibroblasts and enhance the hair-inducing capacity of fibroblasts. Specifically, fibroblasts treated with DPC-Exos could induce hair follicle neogenesis on the dorsal of nude mice when co-administered with neonatal mouse epidermal cells. In addition, our findings confirm that DPC-Exos can expedite wound healing and stimulate hair follicle regeneration by activating the Wnt/β-catenin signaling pathway. This study offers a theoretical foundation for the potential therapeutic application of DPC-Exos in wound healing and offers a novel therapeutic approach for regenerative wound healing.

## Data Availability

No datasets were generated or analysed during the current study.
